# [Retracted] A study of Sirt1 regulation and the effect of resveratrol on synoviocyte invasion and associated joint destruction in rheumatoid arthritis

**DOI:** 10.3892/mmr.2026.13986

**Published:** 2026-08-10

**Authors:** Liang Hao, Yuying Wan, Juhua Xiao, Qiang Tang, Huan Deng, Lu Chen

Mol Med Rep 16: 5099–5106, 2017; DOI: 10.3892/mmr.2017.7299

Following the publication of the above paper, it was drawn to the Editor's attention by a concerned reader that western blot data featured in Fig. 5 on p. 5103 were remarkably similar to data that had already been submitted to the same journal, *Molecular Medicine Reports*, in a paper that was written by different authors at different research insititutes, but which has subsequently been retracted. Similarly, western blot data featured in Fig. 2 on p. 5102 were strikingly similar to data that were featured in another paper written by different authors at different research institutes which was submitted to the journal *Biomed Research International* a few months afterwards.

Owing to the fact that the contentious data in the above article had already been submitted for publication prior to its submission to *Molecular Medicine Reports*, the Editor has decided that this paper should be retracted from the Journal. The authors were asked for an explanation to account for these concerns, but the Editorial Office did not receive a reply. The Editor apologizes to the readership for any inconvenience caused.

